# Ubiquitin D is correlated with colon cancer progression and predicts recurrence for stage II-III disease after curative surgery

**DOI:** 10.1038/sj.bjc.6605870

**Published:** 2010-08-31

**Authors:** D-W Yan, D-W Li, Y-X Yang, J Xia, X-L Wang, C-Z Zhou, J-W Fan, Y-G Wen, H-C Sun, Q Wang, G-Q Qiu, H-M Tang, Z-H Peng

**Affiliations:** 1 Department of General Surgery, Shanghai Jiaotong University Affiliated First People's Hospital, 85 Wujin Road, Shanghai 200080, People's Republic of China; 2 Department of General Surgery, Ningxia Medical University Affiliated Hospital, Yinchuan, People's Republic of China; 3 Department of Pathology, Shanghai Jiaotong University Affiliated First People's Hospital, Shanghai, People's Republic of China

**Keywords:** ubiquitin D, colon cancer, progression, prognosis, tissue microarray

## Abstract

**Background:**

Our recent study observed that the expression of ubiquitin D (UBD), a member of ubiquitin-like modifier family, was upregulated in colon cancer parenchymal cells. The present study further investigated the clinical signicance of UBD in colon cancer.

**Methods:**

Using quantitative PCR, tissue microarray (TMA), western blot analysis and immunohistochemical stain, we evaluated UBD mRNA and protein levels in tumour tissues from patients with colon cancer at different stages and in paired adjacent normal epithelium.

**Results:**

Immunohistochemical detection of UBD on a TMA containing 203 paired specimens showed that increased cytoplasmic UBD was signicantly associated with depth of cancer invasion, lymph node metastasis, distant metastasis, tumour histologic grade, advanced clinical stage and Ki-67 proliferative index. Patients with UBD-positive tumours had a significantly higher disease recurrence rate and poorer survival than patients with UBD-negative tumours after the radical surgery. Stratification analysis according to tumour stage revealed UBD as an independent predictor for tumour recurrence in patients with stage II and III tumours.

**Conclusion:**

UBD may contribute to the progression of colon carcinogenesis and function as a novel prognostic indicator of forecasting recurrence of stage II and III patients after curative operations.

Colon cancer is a major cause of cancer morbidity and mortality and is the third most fatal malignancy worldwide (Jemal *et al*, 2009). In China and other economically transitioning countries, colon cancer incidence rates have increased over the last 20 years, most likely due to changes in the environment and individual life style and nutritional habits (Zhang *et al*, 2009). In certain high prevalence regions, colon cancer mortality has become the second leading cause of cancer death (Jiang *et al*, 2009). Surgical resection is the most widely used treatment for colon cancer. tumour recurrence, the main factor for the failure of colon cancer therapy following radical surgery, negatively impacts patient quality of life and frequently results in patient mortality. At present, risk assessment for colon cancer recurrence is mainly based on tumour node metastasis staging (O’Connell *et al*, 2004). However, clinical outcomes are quite variable, even among patients diagnosed at the same tumour stage (Galandiuk *et al*, 1992). Therefore, there is an urgent need to discover and utilize novel factors for predicting tumour recurrence at the time of operation that could assist in implementing individualized, directed therapeutic regimens.

The complicated process of tumour recurrence involves a number of biological changes, including deregulated expression of several oncogenes and tumour suppressor genes within tumour cell subpopulations (Grady and Carethers, 2008). Although several molecular biomarkers, including K-ras, p53 and DPC4, have been evaluated as candidate prognostic indicators in colon cancer (Paul-Samojedny *et al*, 2005; Duffy *et al*, 2007), none of these markers has been widely adopted due to conicting literature reports. The protein Ki-67 (also known as MKI67) is a cellular marker for proliferation that is detectable within the cell nucleus during all active phases of the cell cycle (G1, S, G2 and mitosis), but is not expressed in resting cells (Scholzen and Gerdes, 2000). Although Ki-67 expression is widely used as a tumour proliferative index and has been associated with colon cancer treatment response and prognosis (Salminen *et al*, 2005; Fluge *et al*, 2009), its clinical utility in predicting disease outcomes remains a topic of investigation.

We recently reported the use of laser capture microdissection and complementary  DNA microarrays to explore gene expression profiles in colon cancer parenchymal cells (Fan *et al*, 2008). Using these techniques, we showed upregulation of ubiquitin D (UBD, also known as FAT10) levels in cancer cells (signal log ratio 1.9) when compared with normal colon epithelial tissue (paper in preparation). UBD was first discovered in reticuloendothelial tissues and mucosal-associated lymphoid immunological systems as one of the genes at the human major histocompatibility complex class I locus on chromosome 6 (Fan *et al*, 1996; Bates *et al*, 1997; Ebstein *et al*, 2009). The UBD gene encodes an 18 kDa protein containing an N- and C-terminus with 29 and 36% identity with ubiquitin, respectively (Fan *et al*, 1996). Of the ubiquitin-like proteins that have been identified, UBD is the only one of that conjugate to target proteins by a free diglycine motif at the C-terminus and directly guides noncovalently bound proteins to proteasomal degradation (Hipp *et al*, 2005; Kalveram *et al*, 2008; Schmidtke *et al*, 2009).

UBD also has important roles in cell mitosis, chromosome instability, apoptosis and immune response (Raasi *et al*, 2001; Canaan *et al*, 2006; Lim *et al*, 2006; Ren *et al*, 2006). UBD deregulation may induce abnormal alterations in apoptosis, cell division or chromosome instability, which are associated with neoplastic change (Sarasin, 2003; Adler *et al*, 2009). tumour UBD expression shows some tissue specificity, with transcriptional upregulation observed in liver, uterine cervix, ovarian, pancreatic, gastric and small intestine adenocarcinomas, but not in thyroid, prostate or kidney cancers (Lee *et al*, 2003; Lukasiak *et al*, 2008a). In hepatic cancer cells, increased expression of UBD was associated with Proliferating Cell Nuclear Antigen, a cell proliferation marker, and reported to provide a growth advantage over cells without UBD expression (Oliva *et al*, 2008). High UBD expression also promoted hepatocellular carcinoma development in a mouse model and formation of Mallory–Denk bodies, which are preneoplastic changes in chronic liver disease (Oliva *et al*, 2008, 2009). Overexpression of UBD in gastric cancer has been correlated with metastasis and tumour staging, and both UBD mRNA and protein levels were identified as independent prognostic factors for this disease (Ji *et al*, 2009). Increased UBD has also been positively correlated with mutant p53 expression, which may activate UBD expression and indirectly facilitate gastric cancer progression (Zhang *et al*, 2006; Ji *et al*, 2009). Interferon-*γ* and tumour necrosis factor-α act synergistically to induce the UBD promoter through an interferon sequence resposive element (Oliva *et al*, 2010). Collectively, these data indicate that UBD may be a marker for precancerous lesions and may promote cancer progression.

The clinical significance and prognostic value of UBD expression in colon cancer has not been reported. The present study evaluated UBD expression in fresh frozen colon cancer specimens and paired normal epithelium and cancer samples on a tissue microarray (TMA) to establish whether UBD expression was associated with the clinicopathological features of colon cancer, risk of disease recurrence or patient survival.

## Materials and methods

### Tissue specimens

A total of 203 patients with colon cancer permitted operation by the same surgical team with the General Surgery Department of Shanghai Jiao Tong University Affiliated First People's Hospital from 2001 to 2003. No patients received either chemotherapy or radiotherapy before surgery. There were 86 male and 117 female patients, with a median age of 68 years (range, 22–95 years) at the time of operation. The tumour grade and stage classification were made as previously described (O’Connell *et al*, 2004; Chen *et al*, 2009). Patients with stage III and IV disease underwent standard chemotherapeutic protocols with 5-fluorouracil following surgery as per The National Comprehensive Cancer Network Practice Guidelines for Colorectal Cancer (Benson *et al*, 2000). Depending on the disease status, one of the three different therapy plans was used on each patient. Although most of the patients began to receive chemotherapy 2 weeks after the surgery, some patients received the chemotherapy 3–4 weeks after the surgery on the basis of their recovery. Supplementary Table S1 describes the post-operative chemotherapy plan for patients with stage III or IV colon cancer.

Detailed patient demographic information is presented in Table 1. Vascular invasion was defined as vessel wall occlusion or destruction, with a surrounding fibroinflammatory reaction (Chen *et al*, 2009). The median patient follow-up time was 61 months after surgery (range, 9–89 months). All patients provided informed consent according to a protocol approved by the Institutional Review Board of Shanghai First People's Hospital.

### RNA extraction, quantitative real-time PCR and reverse transcription PCR

Total RNA was extracted from frozen primary tumour and adjacent normal mucosa of colon cancer specimens according to the manufacturer's instructions (Qiagen, Hilden, Germany), and then 1 *μ*g RNA was reverse transcribed into complementary DNA using an A3500 RT-PCR System (Promega Corporation, Madison, WI, USA). The primers used for RT-PCR were: UBD, sense 5′-CATCCACCTTACCCTGAA-3′ and antisense 5′-ATACCCGTCTTAGTCTCG-3′ (156 bp); and *β*-actin, sense 5′-CGGGAAATGTGCGTGAC-3′ and antisense 5′-TGGAAGGTGGACAGCGAGG-3′ (434 bp). A 1 *μ*l aliquot of complementary DNA was amplified using specific primers and GoTaq Green Master Mix M7122 (Promega) in a MJ PTC-100 programmable thermal controller (Bio-Rad, Hercules, CA, USA). The cycling conditions were as follows: 95°C for 2 min, and then 30 cycles of 95°C for 30 s, 55°C for 30 s and 72°C for 45 s, with a final extension at 72°C for 5 min. PCR products were separated on 1.5% agarose gels and then visualized using an ultraviolet imaging system (FuRi Co., ShangHai, China).

To further confirm *UBD* gene expression in colon tumours, relative UBD mRNA levels were assessed in 30 randomly selected, paired normal mucosa and colon cancer tissues using an ABI Prism 7500 quantitative real-time PCR (qPCR) system (Applied Biosystems, Foster City, CA, USA) and the IQTM SYBR Green Supermix Kit (Bio-Rad) according to manufacturer's instructions and using the thermal cycling conditions described above. The primers for qPCR were: UBD, sense 5′-TTGATGCCAACCCATATGACAG-3′ and antisense 5′-ATACCCGTCTTAGTCTCG-3′ and glyceraldehyde 3-phosphate dehydrogenase, sense 5′-TGACTTCAACAGCGACACCCA-3′ and antisense 5′-CACCCTGTTGCTGTAGCCAAA-3′. Each reaction was repeated at least three times, and then the mean UBD mRNA level for each tumour was compared with the level its matched, non-tumourous tissue. The fold change (2-^ΔΔ^Ct) in UBD expression in each paired sample was calculated using the formulas: UBD^Δ^Ct=(Avg.UBD_Ct−Avg.GAPDH_Ct), UBD^ΔΔ^Ct=(UBD^Δ^Ct_tumour−UBD^Δ^Ct_non-tumour).

### Western blot

Total protein was extracted from frozen colon tumour and adjacent normal tissue samples using an ice-cold Radio Immunoprecipitation Assay lysis buffer (50 mM Tris pH 7.4, 150 mM NaCl, 1% NP-40, 0.5% sodium deoxycholate and 0.1% sodium dodecyl sulphate). Protein concentrations were measured using a BCA protein assay kit (Beyotime Biotechnology Co., Jiangsu, China). Equivalent amounts of protein were separated on 12% sodium dodecyl sulphate–polyacrylamide gels and then transferred onto polyvinylidene difluoride membranes. The membranes were blocked in 5% fat-free milk with 0.1% Tween 20 for 1 h at room temperature, followed by incubation with either the UBD primary mouse polyclonal antibody (1 : 500, H00010537-B01, Abnova, Taipei, China) or *β*-actin antibody (1 : 1000, Cell Signaling Technology, Beverly, MA, USA) overnight at 4°C. After washing with TBST buffer (10 mM Tris pH 8.0, 150 mM NaCl, 0.1% Tween-20), blots were incubated with a goat-anti-mouse immunoglobulin G–horseradish peroxidase conjugate secondary antibody (1 : 4000, Abnova) for 40 min, and then bound antibodies were detected using enhanced chemiluminescence (Pierce Biotechnology, Rockford, IL, USA) and exposure to X-ray film. The abundance of each protein was determined and normalized against *β*-actin expression.

### TMA construction

For TMA construction, formalin-fixed, paraffin-embedded samples containing primary tumours and paired normal mucosa, among them 66 specimens of paired metastatic lymph nodes, were retrieved from archives of the Department of Pathology of our Hospital. Representative areas of tissue were established by microscopic review of H&E stained slides and 2.0 mm diameter cores were punched from the paraffin blocks. Two cores from each of primary cancer and normal tissues at a distance of at least 2 cm from the tumour were arrayed. TMAs were created using a Tissue Microarrayer (Beecher Instruments, Sun Prairie, WI, USA). All specimens were examined by at least two pathologists to prevent bias. Tumour and normal mucosa morphology on the arrays were validated as having high accordance with that of the whole archived section.

### Immunohistochemistry

UBD and Ki-67 expression were detected on the TMAs following citrate buffer (pH 6.0) antigen retrieval using standard methodology and a primary antibody against UBD (1 : 200, Abnova) or Ki-67 (1 : 50, Dako Cytomation, Copenhagen, Denmark). Tissue sections were counterstained with Mayer's hematoxylin. Positive staining was scored by two independent investigators without the knowledge of patient outcomes (double-blinded) according to the staining area and intensity as described by (Bachmann *et al*, 2008). Staining intensity was graded as follows: 0, no staining; 1+, mild staining; 2+, moderate staining; and 3+, intense staining. The staining area was scored using the following scale: 0, no staining of cells; 1+, <10% of tissue stained positive; 2+, 10–50% stained positive; and 3+, >50% stained positive. The sum of staining score (intensity + extension) index was designated as follows: 0–2, negative expression; 3–4, weak expression; and 5–6, strong expression. Analysis of Ki-67 index was on the basis of the percentage of nuclei stained positive for Ki-67. We selected 10% positively staining nuclei as the cutoff point dividing the negative group (⩽10% cells with positive nuclei) and the positive group (>10% cells with positive nuclei) (Hashimoto *et al*, 2006). When discrepancy in an assessment was encountered, the slides were re-examined by both pathologists under a multi-head microscope to obtain an agreement.

### Statistical analysis

For continuous variables, data are expressed as the median and inter-quartile range and compared using the Mann–Whitney *U*-test for two-group comparisons or the Kruskal–Wallis test for three groups. For categorical variables, data are expressed as the numerical count and percentage and compared using the Fisher's exact test. McNemar's and Wilcoxon signed rank tests were used to compare the dependent categorical (UBD staining for the 66 matched specimens in lymph node metastasis (LNM) and primary tumour) and continuous (real-time PCR analysis of UBD in 30 paired tumour tissues and adjacent normal mucosa) variables, respectively. Survival curves were calculated using the Kaplan–Meier method. A log-rank test was used to compare the survival curves. If the data violated the proportional hazard assumption, the Breslow test was performed instead of the log rank test. Cox proportional hazard models were used to investigate the independent risk factors for death and lymph nodes metastasis. Significant factors in univariate Cox proportional hazard models were selected for the final multivariate regression model using the forward conditional method. All statistical analyses were set with a significance level of 0.05 and performed using SPSS 15.0 statistical software (SPSS Inc., Chicago, IL, USA).

## Results

### Upregulation of UBD expression in primary colon cancer as compared with adjacent normal mucosa

**Figure 1:**
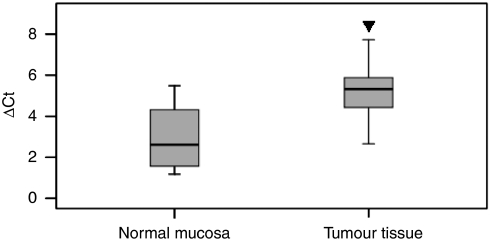
Real-time PCR analysis of UBD mRNA expression in 30 paired colon tumour samples and adjacent normal mucosa. For each sample, the relative UBD mRNA level was normalized using *β*-actin expression. Data are presented as the median (line) ΔCt value with boxed 25th and 75th percentiles. The data range is represented by the upper and lower bars. ▾*P*<0.001.

Of the 30 randomly selected, paired cases used for evaluating UBD mRNA and protein expression, 22 (73%) colon cancers showed at least a two-fold increase in UBD mRNA level as compared with that of the adjacent, non-cancerous tissue. This difference in UBD mRNA expression was significant (*P*<0.001, Figure 1). Subsequent RT-PCR and western blotting confirmed that both UBD mRNA and protein levels were upregulated in cancerous tissues as compared with adjacent normal mucosa (Supplementary Figure S1).

### Association of UBD TMA immunohistochemical staining with patient clinicopathological parameters

**Figure 2:**
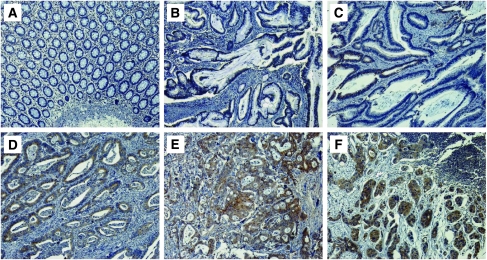
Immunohistochemical staining of Ubiquitin D (UBD) expression in normal tissue and colon cancer. (**A**, **B**) Negative UBD expression in normal colonic epithelium (**A**) and well-differentiated tumour (**B**). (**C**) Weak, focal cytoplasmic UBD staining in a well-differentiated colon tumour. (**D**, **E**) Diffuse, intense UBD staining in moderately (**D**) and poorly (**E**) differentiated colon tumours. (**F**) Strong UBD staining in a colon cancer lymph node metastasis. Original magnication × 100.

Of the 203 normal mucosa specimens on the paired TMA, 191 (94%) showed negative UBD expression, with weak staining in 6 (3%) cases and strong staining in 6 (3%) additional cases. In contrast, UBD expression was obvious in the majority of colon tumour specimens, with weak staining in 71 (35%) cases, strong staining in 64 (31%) cases and negative staining in 68 (33%) cases. UBD was predominantly localized in the cytoplasm of colonic epithelial and tumour cells, with nuclear staining only rarely observed (Figure 2).

Among the 18 stage IV cases, only one patient who had received noncurative metastatic resection and who was excluded from the survival analysis, showed negative UBD staining in the primary tumour. Associations between clinicopathological factors and UBD expression are summarized in Table 1. Increased UBD expression was signicantly correlated with depth of tumour invasion (pT stage), LNM (pN stage), distant metastasis (M stage,), histologic grade (Figure 2B–E) and advanced American Joint Committe on cancer (AJCC) stage. No correlations were found between UBD expression and age, gender, tumour location or vascular invasion.

The paired colon tumour and normal mucosa TMA was also stained for Ki-67 expression. The median Ki-67 staining percentage in the colon series was 25% (range, 5–80%). The frequency of Ki-67 immunopositivity was 79% (160/203). Ki-67-positive staining was signicantly associated with tumour pT stage and advanced patient clinical stage (Table 1). Also, Ki-67-positive staining was more frequent in cases with weak and strong UBD staining than with cases with negative UBD staining (37% weak *vs* 35% strong *vs* 27% negative; *P*=0.001), suggesting a statistical correlation between UBD expression and Ki-67. Indeed, a co-expression pattern between UBD and Ki-67 staining was often observed in the same area of tumour tissues (Supplementary Figure S2).

It is worth noting that strong UBD staining was observed in metastatic colon cancer cells within positive lymph nodes (Figure 2F). In the 66 matched specimens available for analysis, the rate of positive UBD expression in colon cancer lymph node metastatic cells was higher than in the paired primary tumours (55/66, 83% *vs* 46/66, 69% *P*=0.002, Table 2). These data suggest that upregulated UBD expression may correlate with colon tumour metastasis.

A total of 78 of the 195 (40%) patients who underwent curative operations experienced disease relapse. There were 56 patients (56/78, 71%) with recurrent disease within 36 months of surgery (median 28 months, range 4–65 months). In this cohort, there was an obviously signicant correlation between UBD expression and total tumour recurrence. Patients with weak and strong UBD staining in primary tumours had higher recurrent rates than did patients with negative primary tumour staining (weak 38/68, 55% strong 26/60, 43% negative 14/67, 20% *P*=0.006). Positive (weak and strong) UBD expression was strongly associated with an increased risk of tumour recurrence as compared with negative UBD expression (RR 3.786; 95% CI 1.912–7.497; *P*<0.001). However, there was no significant relationship detected between UBD level of expression and time to onset of recurrent disease (negative, range 6–65 months; weak, 4–58 months; strong, 5–64 months; *P*=0.056).

### Survival analysis and prognostic significance of UBD expression according to AJCC stage stratification

**Figure 3:**
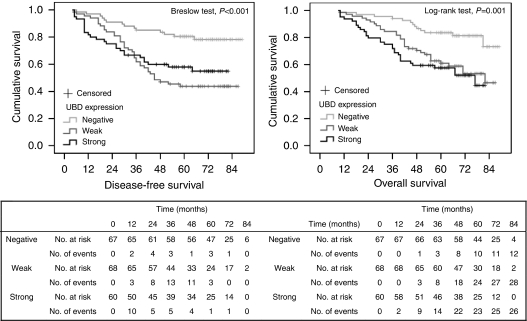
Kaplan–Meier plots of disease-free survival (left) and overall survival (right) of patients with colon cancer who underwent curative resections on the basis of the immunohistochemical UBD expression.

To avoid the potential confounding influences of unresectable metastatic tumours and additional patient post-operative management on the results of these analyses, eight of the patients with stage IV disease and noncurative surgery were excluded from the survival analyses. At the end of the study, 66 of 195 (33%) patients had died from their disease and 129 patients were still alive. Patients with negative tumour UBD expression had a better 5-year disease-free survival (DFS) and overall survival (OS) rate than did the group with positive UBD expression (DFS: 83% negative *vs* 57% positive; OS: 85% *vs* 63% *P*=0.001, respectively). Kaplan–Meier curves showed that the rate of recurrence was signicantly elevated with positive UBD expression (Figure 3). The mean relapse-free survival times for patients with negative and positive UBD expression were 77.90±2.98 and 60.22±2.85 months, respectively (*P*=0.001). The OS rate was also signicantly decreased with increasing UBD expression (Figure 3). By univariate analysis (Table 3), both increased post-operative recurrence and decreased OS were associated with pT stage, pN stage, AJCC stage, vascular invasion, Ki-67 expression and UBD expression. Multivariate analysis (Table 3) demonstrated that positive tumour UBD expression remained a signicant independent prognostic factor for increased disease recurrence and decreased survival.

To further define increased UBD expression as an independent factor influencing tumour recurrence irrespective of clinical disease stage, an analysis was performed with adjustment for AJCC stage stratification. No significant difference was found in DFS between the negative and positive UBD expression groups of patients with stage I disease (Supplementary Figure S3*A*). Two patients with negative UBD staining died, which may need to be explained by future research with an expanded sample set. However, significant differences in DFS were detected in patients with stage II and III disease. The 5-year DFS rates in stage II patients of negative and positive UBD expression were 100 and 77.3%, respectively (Supplementary Figure S3*B*), and in stage III were 64 and 38%, respectively (Supplementary Figure S3*C*). Of the 10 patients with stage IV disease and positive tumour UBD staining, all experienced tumour recurrence within 3 years after radical resection. These data indicate that UBD levels are with a shorter relapse-free duration that was independent of tumour staging.

## Discussion

This is the first report showing the association of upregulated human colon cancer UBD expression with cancer progression and recurrence independent of Pathological Tumour-Node-Metastasis staging. These data support UBD as a novel prognostic indicator of colon cancer outcomes, in particular for forecasting recurrence in patients with stage II and III disease following curative surgery. Correlations of UBD expression with cell proliferation and advancing tumour stages suggests that UBD may contribute to the progression of colon carcinogenesis.

UBD is a unique member of ubiquitin-like modifier family, first found during mapping of the *HLA-F* gene (Fan *et al*, 1996). Upregulation of UBD mRNA has been observed in hepatocellular, gastrointestinal and gynecological carcinomas (Lee *et al*, 2003) and expression of both UBD mRNA and protein has been verified in fibroblasts and hepatic cancer cells (Raasi *et al*, 2001; Lukasiak *et al*, 2008a). In the present study, we first measured UBD expression in fresh frozen specimens of colon cancer and found that UBD mRNA and protein expression levels were higher in colon tumour tissue than in the surrounding noncancerous mucosa. These data indicated that UBD was upregulated at both transcriptional and post-transcriptional levels. Further validation by immunohistochemistry showed that 66% (135/203) of primary colon cancers had positive UBD protein staining, whereas only 6% (12/203) of normal colonic epithelium were immunoreactive for UBD. These data suggest that UBD might have an important role in the progression of colon carcinogenesis. UBD is reported to have important roles in the regulation of cell mitosis, chromosome instability, apoptosis and immune response (Raasi *et al*, 2001; Canaan *et al*, 2006; Lim *et al*, 2006; Ren *et al*, 2006).

UBD was reported to be localized in the nuclei of hepatocellular and gastric cancer cells, indicating that it might mediate transcriptional control and tumour development (Lee *et al*, 2003; Ji *et al*, 2009). However, in the present study, nuclear localization of UBD was only rarely observed. It has been reported that UBD was closely related to gastric cancer LNM and advanced tumour node metastasis stages (Ji *et al*, 2009). However, there are currently no published reports on the possible association of UBD expression with the clinicopathological features of colon cancer. Our results revealed significant correlations between tumour UBD overexpression and clinical stage, pT, pN stage, distant metastasis and tumour histological grade. These strong correlations suggest that UBD overexpression might promote tumour invasion and metastasis, and that UBD could possibly be used a biomarker for identification of subsets of colon cancer with a more aggressive phenotype. Our data also showed a significant association between tumour UBD expression and Ki67 index, suggesting that UBD may be involved in the increased proliferation of colon cancer cells.

It has been suggested that UBD expression may be related to other biomarkers used to predict tumour metastasis, such as CD44v6, nm23, MTA1 and matrix metalloproteases (Ji *et al*, 2009). Our data revealed that UBD levels were higher in tumours with associated LNM than those without LNM. Positive UBD protein expression was signicantly higher in metastatic colon cancer cells within lymph nodes than in matched primary tumours. These data suggested that increased UBD expression might correlate with the invasive behavior and metastatic processes of colon cancer.

The mechanism by which UBD contributes to tumorigenesis is not well elucidated. According to previous reports, UBD is a downstream target of p53 and its promoter is negatively regulated by p53. UBD overexpression in cancers was ascribed to transcriptional upregulation upon the loss of p53 (Zhang *et al*, 2006), but mutations in the UBD coding sequence have not been reported (Lukasiak *et al*, 2008b). The proinammatory cytokines interferon-*γ* and TNF-*α* can induce UBD expression in conjunction with an immune response within the tumour microenvironment (Lukasiak *et al*, 2008b). Excessive UBD protein might bind noncovalently to spindle-assembly checkpoint protein MAD2 (Liu *et al*, 1999; Mapelli *et al*, 2007), resulting in the inhibition of MAD2 function during the prometaphase stage of the cell cycle and a reduction in cell-cycle time (Lim *et al*, 2006). These changes could lead to genomic instability and tumour formation (Michel *et al*, 2001; Ren *et al*, 2006; Adler *et al*, 2009). In addition, UBD may influence caspase-dependent apoptosis in HeLa and human renal tubular epithelial cells (Raasi *et al*, 2001; Ross *et al*, 2006). Further experimentation is required to define the molecular mechanisms governing the potential role UBD expression in colon cancer progression.

Until now, valid prognostic biomarkers for colon cancer have not been established (Grady and Carethers, 2008). In the present study, patients with a high tumour UBD expression had an increased risk of tumour recurrence and shorter survival. It is interesting that the patients of stage II and III disease and tumours with positive UBD expression were at greater risk for tumour recurrence. The 5-year DFS rates for the negative and positive UBD expression groups were 93 and 80%, respectively, for stage II disease and 70.8 and 36%, respectively, for stage III disease. Although all 10 patients with stage IV disease who were included in the survival analysis had positive tumour UBD expression and post-operative disease recurrence, a statistical relationship could not be established owing to the small sample size. Although lymph node status is different between stage II and III disease and LNM is usually regarded as a poor prognosis factor for colon cancer, whether lymph node involvement should be routinely assessed remains a topic for debate (Johnson *et al*, 2006; Wang *et al*, 2008). Also, the current AJCC classification of stage III represents a heterogeneous patient population (Johnson *et al*, 2006). The present study has delineated the potential utility of assessing UBD expression for predicting recurrence of stage III (lymph node-positive) patients. This information could contribute to clinical decisions and help target drastic therapeutics to patient subgroups with a higher likelihood of disease recurrence and metastasis. Study limitations include the small number of patients with relatively short follow-up time. As increased UBD expression in tumour cells is a phenotypic change that indicates a preneoplastic change, it will be interesting to include colon adenomas with or with out dysplasia or intra mucosal carcinoma in future studies to validate our results showing that increased UBD expression in colon cells may indicate a preneoplastic change.

To the best of our knowledge, this is the first report to show the clinical signicance of tumour UBD expression in colon cancer. UBD was overexpressed in tumour tissue and was associated with aggressive colon cancer phenotypes. We propose that tumour UBD expression may be a clinically useful, prognostic indicator of poor patient survival, independent of tumour stage. Overexpression of UBD in stage II and III colon cancer may correlate with disease recurrence. These preliminary findings need to be verified in a larger, prospective, controlled clinical study.

## Figures and Tables

**Table 1:** Association between clinicopathological features and UBD or Ki-67 protein expression

	**UBD expression**				**Ki-67 index**		
	**Negative (*n*=68)**	**Weak (*n*=71)**	**Strong (*n*=64)**	***P***	**Negative (*n*=43)**	**Positive (*n*=160)**	***P***
Age (mean, range)	69.5 (54.0, 74.0)	66.0 (58.0, 75.0)	71.0 (57.5, 78.0)	0.208	71.0 (62.0, 78.0)	67.0 (56.0, 75.0)	0.132
							
*Gender (n*, %)							
Male	34 (50.0%)	28 (39.4%)	24 (37.5%)	0.289	18 (41.9%)	68 (42.5%)	1.000
Female	34 (50.0%)	43 (60.6%)	40 (62.5%)		25 (58.1%)	92 (57.5%)	
							
*Location (n*, %)							
Right	29 (42.6%)	25 (35.2%)	30 (46.9%)	0.594	14 (32.6%)	70 (43.8%)	0.139
Transverse	6 (8.8%)	6 (8.5%)	7 (10.9%)		7 (16.3%)	12 (7.5%)	
Left	33 (48.5%)	40 (56.3%)	27 (42.2%)		22 (51.2%)	78 (48.8%)	
							
*T Stage (n*, %)							
T1	3 (4.4%)	2 (2.8%)	3 (4.7%)	0.027^*^	3 (7.0%)	5 (3.1%)	<0.001^*^
T2	15 (22.1%)	6 (8.5%)	2 (3.1%)		15 (34.9%)	8 (5.0%)	
T3	20 (29.4%)	27 (38.0%)	29 (45.3%)		11 (25.6%)	65 (40.6%)	
T4	30 (44.1%)	36 (50.7%)	30 (46.9%)		14 (32.6%)	82 (51.3%)	
							
*N stage (n*, %)							
N0	43 (63.2%)	37 (52.1%)	28 (43.8%)	0.021^*^	27 (62.8%)	81 (50.6%)	0.170
N1	21 (30.9%)	18 (25.4%)	22 (34.4%)		8 (18.6%)	53 (33.1%)	
N2	4 (5.9%)	16 (22.5%)	14 (21.9%)		8 (18.6%)	26 (16.3%)	
							
*M stage (n*, %)							
M0	67 (98.5%)	62 (87.3%)	56 (87.5%)	0.018^*^	41 (95.3%)	144 (90.0%)	0.374
M1	1 (1.5%)	9 (12.7%)	8 (12.5%)		2 (4.7%)	16 (10.0%)	
							
*AJCC stage (n*, %)							
I	14 (20.6%)	7 (9.9%)	3 (4.7%)	0.019^*^	15 (34.9%)	9 (5.6%)	<0.001^*^
II	28 (41.2%)	28 (39.4%)	25 (39.1%)		12 (27.9%)	69 (43.1%)	
III	25 (36.8%)	27 (38.0%)	28 (43.8%)		14 (32.6%)	66 (41.3%)	
IV	1 (1.5%)	9 (12.7%)	8 (12.5%)		2 (4.7%)	16 (10.0%)	
							
*Differentiation (n*, %)							
High	99 (48.8%)	46 (56.8%)	53 (43.4%)	<0.001^*^	26 (60.5%)	73 (45.6%)	0.138
Moderate	74 (36.5%)	21 (25.9%)	53 (43.4%)		14 (32.6%)	60 (37.5%)	
Low	30 (14.8%)	14 (17.3%)	16 (13.1%)		3 (7.0%)	27 (16.9%)	
							
*Vascular invasion (n*, %)							
Yes	3 (4.4%)	5 (7.0%)	6 (9.4%)	0.575	1 (2.3%)	13 (8.1%)	0.309
No	65 (95.6%)	66 (93.0%)	58 (90.6%)		42 (97.7%)	147 (91.9%)	
							
*Ki-67 index (n*, %)							
Negative	24 (35.3%)	11 (15.5%)	8 (12.5%)	0.003^*^			
Positive	44 (64.7%)	60 (84.5%)	56 (87.5%)				

Abbreviations: AJCC=American Joint Committe on cancer; UBD=ubiquitin D. ^*^P<0.05 indicates a significant association between variables.

**Table 2:** UBD immunohistochemical staining for protein expression in matched primary colon cancer and lymph node metastases

**Lymph node**	**Primary colon cancer**				
**metastases**	**Negative**	**Weak**	**Strong**	**Total**	***P*-value**
Negative	4 (6.%)^a^	5 (7.56%)	2 (3.%)	11 (16.7%)	0.002^b^
Weak	1 (1.5%)	3 (4.5%)	8 (12.1%)	12 (18.2%)	
Strong	15 (22.7%)	15 (22.7%)	13 (19.7%)	43 (65.2%)	
Total	20 (30.3%)	23 (34.9%)	23 (34.8%)	66 (100.%)	

a *n* (%).

b Significant difference in protein expression by McNemar's test.

**Table 3:** Univariate and multivariate Cox proportional hazard models for overall survival and disease-free survival

	**Overall survival**				**Disease-free survival**			
	**Univariate**		**Multivariate**		**Univariate**		**Multivariate**	
	**HR (95% CI)**	P*	**HR (95% CI)**	P*	**HR (95% CI)**	P*	**HR (95% CI)**	P*
Age	1.00 (0.98,1.02)	0.834			1.00 (0.98, 1.02)	0.952		
								
*Gender*								
Male	—				—			
Female	1.19 (0.72,1.94)	0.500			1.14 (0.72, 1.78)	0.581		
								
*Tumour location*								
Right	—				—			
Transverse	0.80 (0.30,2.09)	0.643			0.83 (0.34, 1.98)	0.669		
Left	1.19 (0.71,1.99)	0.505			1.12 (0.70, 1.79)	0.631		
								
*T stage*								
T1	0.40 (0.10, 1.65)	0.205	0.40 (0.09, 1.80)	0.235	0.34 (0.08, 1.39)	0.132	0.34 (0.08, 1.45)	0.144
T2	0.12 (0.03, 0.49)	0.003	0.32 (0.07, 1.40)	0.131	0.16 (0.05, 0.52)	0.002	0.44 (0.13, 1.53)	0.197
T3	0.36 (0.20, 0.63)	<0.001	0.34 (0.19, 0.61)	<0.001	0.42 (0.26, 0.70)	0.001	0.41 (0.25, 0.70)	0.001
T4	—		—		—		—	
								
*N stage*								
N0	—		—		—			
N1	3.74 (1.96,7.12)	<0.001	2.85 (1.43, 5.68)	0.003	2.73 (1.57, 4.73)	<0.001	1.97 (1.10, 3.52)	0.022
N2	15.02 (7.85,28.75)	<0.001	9.65 (4.60, 20.25)	<0.001	10.22 (5.78, 18.08)	<0.001	6.89 (3.60, 13.17)	<0.001
								
*M stage*								
M0	—		—		—		—	
M1	14.74 (8.15, 26.67)	<0.001	4.74 (2.00, 11.26)	<0.001	9.93 (4.91, 20.07)	<0.001	4.94 (2.29, 10.67)	<0.001
								
*AJCC stage*								
I	—		—		—		—	
II	2.08 (0.47,9.20)	0.336			2.07 (0.61, 6.96)	0.241		
III	9.59 (2.31,39.78)	0.002			6.69 (2.07, 21.58)	0.001		
IV	48.10 (10.15,228.07)	<0.001			37.18 (9.95, 138.97)	<0.001		
								
*Differentiation*								
High	—		—		—		—	
Moderate	2.37 (1.34, 4.18)	0.003	1.24 (0.65, 2.36)	0.514	2.26 (1.35, 3.79)	0.002		
Low	7.50 (4.11, 13.68)	<0.001	2.28 (1.06, 4.88)	0.034	4.87 (2.64, 8.97)	<0.001		
								
*Vascular invasion*								
No	—				—			
Yes	5.22 (2.76,9.86)	<0.001			4.12 (2.16, 7.86)	<0.001		
								
*UBD*								
Negative	—		—		—		—	
Weak	2.62 (1.33,5.15)	0.005	1.33 (0.63, 2.81)	0.453	3.28 (1.78, 6.06)	<0.001	1.82 (0.94, 3.53)	0.075
Strong	3.21 (1.62,6.37)	0.001	2.31 (1.08, 4.93)	0.030	2.64 (1.38, 5.07)	0.003	2.03 (1.03, 3.98)	0.041
								
*Ki-67*								
Negative	—				—			
Positive	2.17 (1.07,4.38)	0.032			2.06 (1.09, 3.89)	0.027	1.88 (0.96, 3.69)	0.065

Abbreviations: AJCC=American Joint Committe on cancer; CI=confidence interval; HR=Hazard ratio; UBD=ubiquitin D.

^*^P<0.05 indicated that the 95% CI of HR was not including 1.

## Appendix

### *Statement of translational relevance*

This is the first report on ubiquitin D association with human colon cancer progression and tumour recurrence independent of Pathological Tumour-Node-Metastasis staging. Although lymph node metastasis and advanced Pathological Tumour-Node-Metastasis stage are widely accepted prognostic indicators for colon cancer (O’Connell *et al*, 2004), methods for assessing lymph node involvement and heterogeneity within tumour classifications remain controversies (Johnson *et al*, 2006; Wang *et al*, 2008). Ubiquitin D was found to predict tumour recurrence in patients with lymph node-negative cancer (stage II), for whom post-operative chemotherapy is only recommended on the basis of the clinical parameters, as there are currently no validated tissue-based biomarkers of disease recurrence. These findings could be clinically translated to help to select patient sub-groups with higher metastatic potential and offer them more appropriate interventions. A major study limitation is the small number of patients with relatively short follow-up time. Larger, prospective controlled studies are required to confirm the present results.
