# Supplementary material for: Ubiquitin D is correlated with colon cancer progression and predicts recurrence for stage II-III disease after curative surgery
Source: Br J Cancer. 2010 Aug 31;103(7):961–9. doi: 10.1038/sj.bjc.6605870 (PMC2965875; doi:10.1038/sj.bjc.6605870)
Supplement: Supplementary Information [file 6605870x1.doc]

**Supplementary data**

**Table S1**. Postoperative chemotherapy plan for patients with stage III or IV colon cancer.

| Regimens | Dose | Administration | Time for drug | Interval cycles | cycles |
| --- | --- | --- | --- | --- | --- |
| **Mayo Clinic Regimen** |  |  |  |  |  |
| Leucovorin (LV) | 20mg/m^2^ | i.v (bolus) | d1-5 | Every 4 wks | six-wks cycles |
| 5-fluorouracil (5-Fu) | 425mg/m^2^ | i.v (bolus) after LV | d1-5 |
| **de Gramont Regimen** |  |  |  |  |  |
| 5-Fu | 400mg/m^2^ | i.v (bolus) | d1+2 | Every 2 wks | six-wks cycles |
| 5-Fu | 600mg/m^2^ | i.v (22h Inf) | d1+2 |
| LV | 200mg/m^2^ | i.v (2h Inf) | d1+2 |
| **FOLFOX4 Regimen** |  |  |  |  |  |
| 5-Fu | 400mg/m^2^ | i.v (bolus) | d1+2 | Every 2 wks | six-wks cycles |
| 5-Fu | 600mg/m^2^ | i.v (22h Inf) | d1+2 |
| LV | 200mg/m^2^ | i.v (2h Inf) | d1+2 |
| oxaliplatin（L-OHP） | 85mg/m^2^ | i.v (2h Inf) | d1 |

i.v.: intravenous. d; day; wks: weeks

**
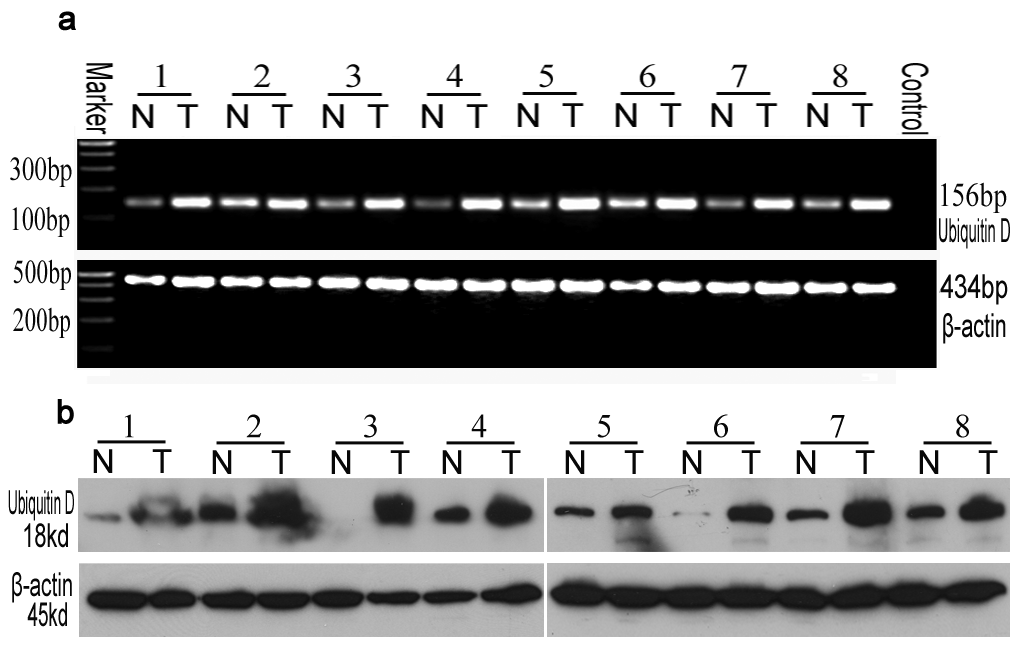
**

**Supplementary Fig. S1.** Representative reverse-transcription PCR (*A*) and Western blot (*B*) results from 8 paired normal (N) and tumor (T) colon tissues. PCR primers and antibodies are described in the text. β-actin expression was assessed as an internal control.

**
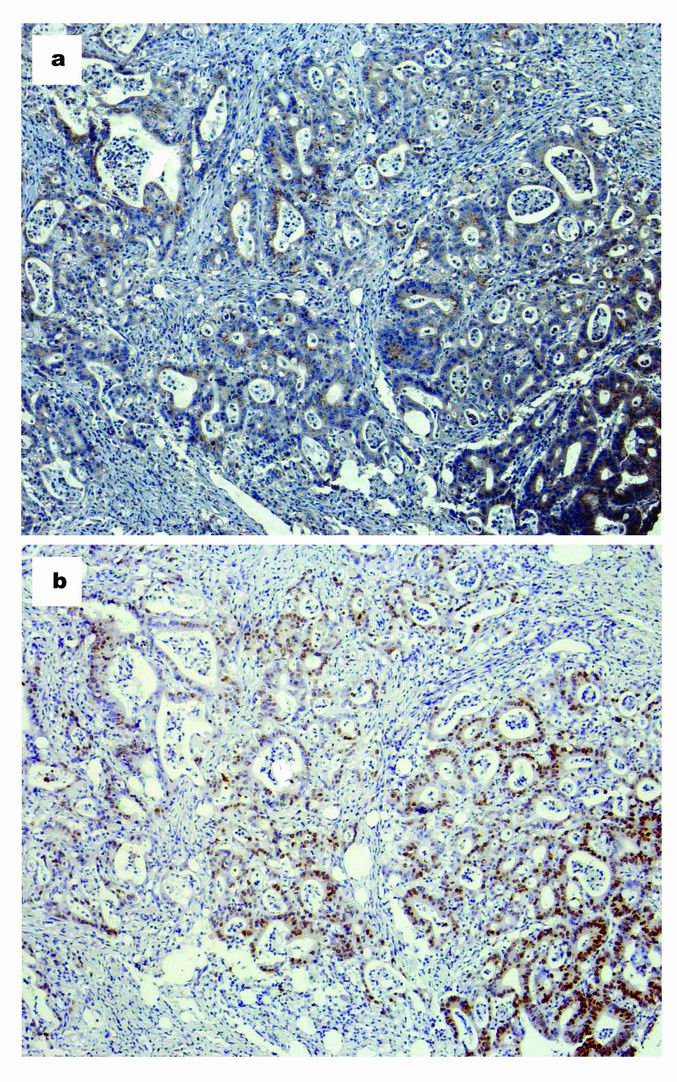
**

**Supplementary Fig. S2.** Coexpression of UBD (*A*) and Ki-67 (*B*) in the same region of a colorectal cancer. A similar trend of increased UBD and Ki-67 expression was observed from the upper left corner to the lower right corner of the tissue specimen. Original magniﬁcation ×100.


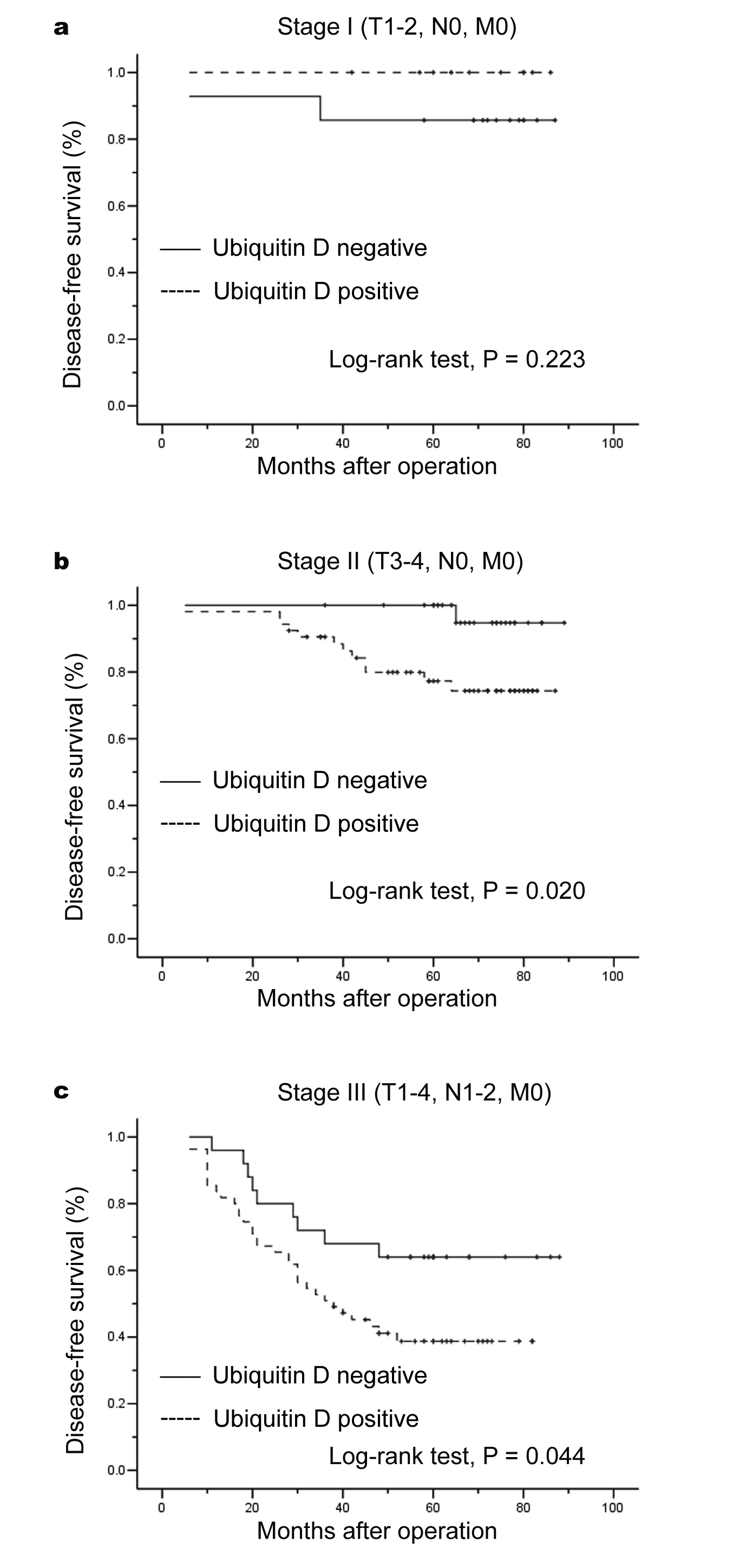


**Supplementary Fig. S3.** Kaplan–Meier plots for disease-free survival analysis of patients with negative and positive UBD expression by AJCC colorectal cancer stage (Stages Ⅰ - Ⅲ).
